# A Case Presentation: Decidualized Endometrioma Mimicking Ovarian Cancer during Pregnancy

**DOI:** 10.1155/2013/728291

**Published:** 2013-04-14

**Authors:** Aybike Tazegül, Özlem Seçilmiş Kerimoğlu, Feyza Nur İncesu, Nasuh Utku Doğan, Setenay Arzu Yılmaz, Çetin Çelik

**Affiliations:** Department of Obstetrics and Gynecology, Faculty of Medicine, Selcuk University, Selcuklu, Konya 42250, Turkey

## Abstract

During pregnancy, masses that are larger than 5 cm and appearing in the Doppler ultrasonography as having increased blood flow, echoes of heterogeneous density, and containing solid components are suspicious for malignancy; however, differential diagnosis of decidualized endometriomas should also be considered. The patient was an 8 weeks pregnant primigravida. The ultrasonographic evaluation showed a cystic mass of size 65 × 57 mm in the left ovary that was well circumscribed, heterogeneous, with highly dense internal echo, and containing a solid component of size 8 × 14 mm. In the 12th week, the ultrasonographic examination revealed an increase in the size of the mass and increased arterial blood flow in the mass. The patient underwent surgery. It was observed that both ovaries were adherent in the Douglas pouch and that the left ovary contained an endometrioma of size 8cm. While the capsule was being peeled, lesions of soft density, with irregular surfaces, and with adhesion in the Douglas pouch were observed. The results of the frozen section revealed decidualized endometrioma and decidual structures. Even in pregnant women when adnexal masses are encountered and the ultrasonography, Doppler, MRI, and CA 125 level analysis still do not favor endometriosis, decidualized endometrioma should be considered in the differential diagnosis.

## 1. Introduction

Diagnostic criteria of endometriosis are the presence of 2 of the following 3 features outside of the uterus: endometrial glands, endometrial stroma, and hemosiderin-laden macrophages. It is a common gynecological problem and it is estimated to affect approximately 6–10% of the women of reproductive age [1]. The macroscopic indicators of the endometriosis can manifest themselves in a variety of ways such as a few petechial, vesicular, hemorrhagic, powderlike implants or serous or clear vesicular structures or intraperitoneal adhesions holding both ovaries, the pouch of Douglas, and uterosacral ligaments; also, in 40–60% of the patients endometriosis is accompanied by ovarian endometrioma [2]. For diagnosis, the imaging method frequently preferred is the vaginal ultrasonography. In 95% of the cases, the sonographic patterns display lesions that are smooth edged, thick capsuled, often with a diameter larger than 10 mm, without papillary proliferations, and containing homogeneous liquid with low echogenicity [3, 4]. Hormonal changes associated with the pregnancy may cause differences in the sonographic appearance of the endometrioma, which results in difficulties in the diagnosis. Fast-growing sonolucent cystic structures with increased blood flow and with intraluminal papillary vegetations are the typical indicators of the malignancies and are also the changes that occur due to decidualized endometrioma [5]. Decidualization is the hypertrophy of the endometrial stroma cells and the development of the decidua formed in response to progesterone to optimally adapt the endometrium for pregnancy [6]. During pregnancy, decidualization can occur outside the uterus, especially in ovarian endometriomas. Deciduosis may also occur in the peritoneal surfaces as a result of subserosal stromal-cell metaplasia due to the effects of progesterone and often disappears after labor as it decreases with decidual involution [7–9]. The case is a primigravida patient, whose decidualized ovarian endometriosis clinically and macroscopically mimicked the symptoms of ovarian malignancy.

## 2. Case Presentation

An 8-week pregnant 32-year-old primigravida patient without prior clinical symptoms of endometriosis applied to the polyclinic with complaints of hyperemesis. The subsequent pelvic ultrasonography showed a cystic mass of size 65 × 57 mm that was well circumscribed, heterogeneous, with internal echo, and containing a solid component of size 8 × 14 mm in the left ovary. CA 125 value was measured as 220. The patient was informed of the suspicion of malignancy and surgery was recommended; however, the patient chose to postpone the operation until the 2nd trimester because of the pregnancy. In the 12th week, the patient was admitted with complaints of left lower-quadrant pain, nausea, and vomiting; the ultrasonographic examination revealed an increase in the size of the mass, 77 × 65 mm, and increased arterial blood flow in the mass. The patient was operated based on her informed consent.

**Figure 1:**
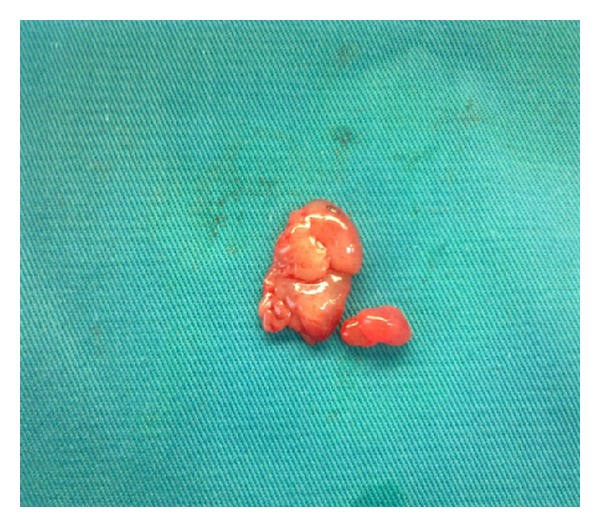
Decidual structures.

**Figure 2:**
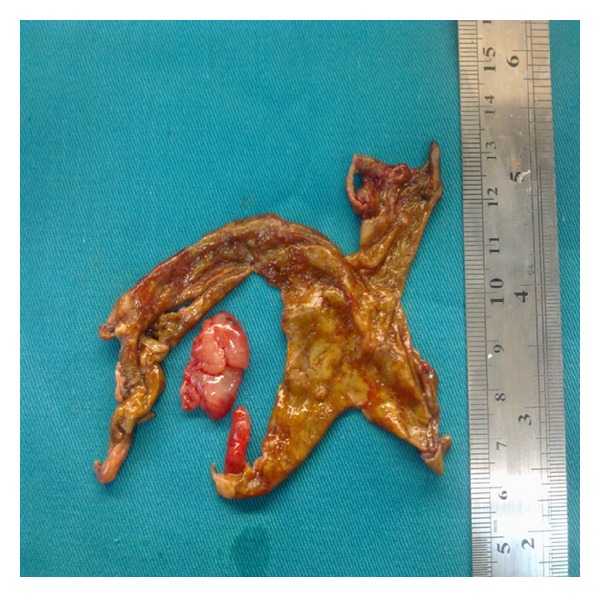
Endometrioma capsule and decidual structure.

**Figure 3:**
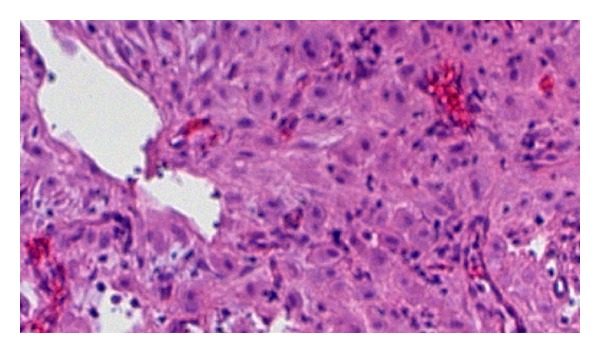
Hematoxylin-eosin stained sections of the left ovary; endometrial glands and decidualized areas are shown.

**Figure 4:**
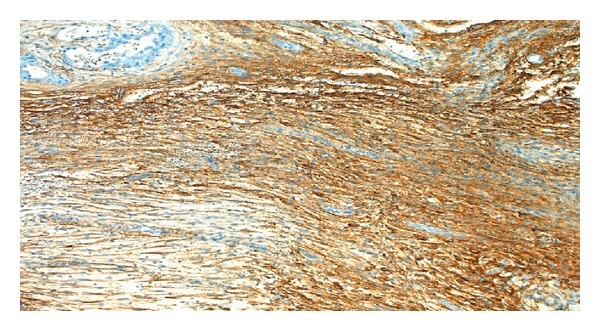
Areas of endometriosis in the ovarian stroma were positively stained with CD10 immune staining.

In the examination of the abdominal cavity, multiple endometriotic foci ‘‘gunpowder burns” on the pelvic side walls, in the pouch of Douglas, and on the intestinal surfaces as well as wide-spread intra-abdominal adhesions were seen. It was observed that both the ovaries were adherent in the Douglas pouch and that the left ovary contained an endometrioma of size 8 cm, whose content was drained during manipulation. While the capsule was being peeled, lesions of soft density, with irregular surfaces, and with adhesion in the Douglas pouch were observed at the base of the capsule that were spreading out of the ovarian capsule, also covering the surfaces of the right ovary and the uterosacral ligaments (Figures 1 and 2). The results of the frozen section showed decidualized endometrioma and decidual structures (Figures 3 and 4). The patient's postoperative care went without any problems; she was injected with 100 mg of micronized progesterone. After the fetal heart rate was checked, the patient was discharged on the 5th postoperative day.

## 3. Discussions 

During pregnancy, decidualization may appear in the endometrial tissues outside the uterus, especially in the endometrial stromal cells of the ovarian endometriomas [6, 7]. Moreover, deciduosis is assumed to be the physiological response of the peritoneal stromal cells to pregnancy; however, it has also been reported to develop in areas such as the appendix, the lymph nodes, the uterine visceral peritoneum, and the cervix [10–14]. It has also been discussed in the literature that in four pregnant patients, the compression caused by the development of a pervasive decidual tissue in the serosa of the appendix results in mechanical obstruction and acute appendicitis [13]. The histological examination of the biopsy material obtained from patients with no prior history of endometriosis detected serosal decidualization rate of 5.5%, and it has been described that the deciduosis in pregnancy is a physiological condition different from endometriosis and that it could be encountered as solid masses, nodules, and even in the form of diffuse subendothelial cell populations [7]. However, having a small number of identified cases of endometrioma decidualization suggests that it is a rare condition. seventeen of the 22 cases described in the literature underwent surgery due to symptoms suggesting malignant ovarian neoplasia, and the histological analysis revealed decidualization of the endometrioma. The study of these cases also discusses adnexal masses that are fast growing, give the impression of malignant in the ultrasonography and MRI, have solid projections, and are detected as increased blood flow by Doppler. However, the presence of septations and acid in the abdomen is mentioned [6, 15, 16]. In one case, high CA 125 levels, abdominal acid, and pleural-effusion complicated bilateral adnexal mass was thought to be an advanced stage ovarian cancer, but was reported as stage 4 endometriosis [17]. A patient in the 19th week of the gestation, who underwent surgery, developed rupture of membrane after the laparotomy and the pregnancy resulted in an abortion [18]. Four cases preferring a conservative approach were observed for a period longer than a year; in the postnatal period, the cyst contents had initially an increased blood flow in the projections, which gradually decreased and disappeared [19]. During the pregnancy of a patient, who was known to have endometrioma before the pregnancy, fast-growing dense-blood-flow papillary projections were identified in the endometrioma; however, in the 10th week an abortion had to be carried out due to a miscarriage and at the end of the following 6 weeks the endometrioma had a normal sonographic reading [20].

In our case, the findings suggestive of malignancy were the size, which was reaching 8 cm, the heterogeneous density and the papillary projections of the echo, and the Doppler ultrasound of the cyst showing the tumor tissue-like increased perfusion. CA 125 levels may also increase by average levels in normal pregnancies. Macroscopically, the presence of papillary projections and its spread out of the cyst capsule towards the pouch of Douglas, the opposite ovarian serosa, and the pelvic side walls pointed to ovarian neoplasia. On the other hand, the presence of adhesions throughout the intra-abdominal, powder-burn lesions on the surfaces of the pelvic side walls and intestinal surfaces, and the image of the cyst contents were findings in favor of the endometrioma. However, ovarian endometriomas may undergo malignant transformation [21, 22]. Therefore, structural changes detected during pregnancy in the ovarian endometrioma should not be ignored.

During pregnancy, although, for the differentiation of the masses in terms of endometrial malignancy, ultrasonography and Doppler findings, CA 125 levels, and the use of MRI are beneficial, when compared to the histological examinations they are insufficient. This case presentation suggests that the lack of ability to clearly carry out the distinction preoperatively necessitates the decidualized endometrial cysts to be included in the differential diagnosis of the pelvic masses during pregnancy.
